# Is the Race Decaying?

**Published:** 1913-01-18

**Authors:** Binnie Dunlop

**Affiliations:** Alexandra Court, S.W.


*To the Editor of* The Hospital.

### Is the Race Decaying?

*To the Editor of* The Hospital.

Sir,—As I have for several years been devoting myself entirely to the study of the important questions which you raise in your article "Is the Race Decaying?" I beg you will allow me the privilege of supplementing it.

The famous trial of Bradlaugh and Mrs. Besant in 1876, for publishing the interdicted pamphlet of Dr. Knowlton, taught the reading public (then mainly the rich) that married people could easily limit their families. The educated classes in all civilised countries immediately began to do so. The population, as the maligned and misrepresented Malthus had predicted, did not diminish—only the deaths did. Although the poor and the unfit continued "propagating their kind unchecked," the falling birth-rates were always accompanied by falling death-rates. These continued to fall steadily owing to the gradual extension downwards of the "knowledge" from the upper classes. The Neo-Malthusians appealed in vain to these classes to teach the poor the importance, and the means, of not bringing more children into the world than they could afford to maintain properly. Hence the reversed selection which you rightly deplore. Only in Holland was this parental prudence freely and openly taught to the classes most in need of it; and there the decline in the general and infantile mortality rates and the increase in the numbers and stature of the inhabitants have been astonishing. The middle classes, too, in that country have not been induced by the burden of public and private charities to restrict their progeny as ours have done.

Instead of our wage-earners each having two or three healthy children, they bring into the world several times that number, half of whom grow up more or less defective, and half of whom die prematurely. If only our upper classes realised the unnecessary suffering and waste caused thereby they would not rest content with the prospect that, as the vital statistics are now progressing, we shall only reach the ideal death-rate (about 10 per 1,000 per annum as in New Zealand) in 1921. Rather would they encourage the doctors to teach the poor mothers, and so make an end of serious poverty, prostitution, degeneration, and social unrest in the next few years. Moreover, the death-rate of 10 per 1,000 with a low birth-rate of 20 per 1,000 would still give us a rapidly increasing population, as was lately the case in Ontario. France's population only increases so slowly because her death-rate continues so high. In conclusion, I would like to say that there are eminent gynæcologists, like Professor Hector Treub, of Amsterdam, who strongly hold that the use of preventives is harmless, and that when the Hungarian Medical Senate was lately asked by their Government to report thereon, it declared them to be not only harmless, but necessary. So why should the great majority of mankind continue to live on the verge of starvation.—I am, Sir, etc.,

Binnie Dunlop, M.B., Ch.B.

Alexandra Court, S.W.,
January 13, 1913.

